# Effects of Ridge-Furrow System Combined with Different Degradable Mulching Materials on Soil Water Conservation and Crop Production in Semi-Humid Areas of China

**DOI:** 10.3389/fpls.2017.01877

**Published:** 2017-10-31

**Authors:** Xiaolong Ren, Xiaoli Chen, Tie Cai, Ting Wei, Yang Wu, Shahzad Ali, Peng Zhang, Zhikuan Jia

**Affiliations:** ^1^College of Agronomy, Northwest A&F University, Yangling, China; ^2^Institute of Water-saving Agriculture in Arid Areas of China, Northwest A&F University, Yangling, China; ^3^Key Laboratory of Crop Physi-Ecology and Tillage Science in Northwestern Loess Plateau, Ministry of Agriculture, Northwest A&F University, Yangling, China

**Keywords:** corn yield, mulching, rainfall harvesting, soil water, soil temperature

## Abstract

In China, the ridge-furrow water conservation planting (RC) system is advantageous for improving crop yields and rainwater use efficiency. In RC planting system, plastic film-mulched ridges are employed for water harvesting while the furrows serve as infiltration and planting belts. To optimize the RC system and to overcome problems due to the lack of water in semi-humid areas at risk of drought, we mulched the furrows with 8% biodegradable film (RC_SB_), liquid film (RC_SL_), or no mulching in the furrows (RC_SN_), while conventional flat planting (CF) was employed as the control. After 4 year (2007–2010) consecutive field study, the results showed that the soil water storage level in the 0–100 cm layer with four treatments was ranked as follow: RC_SB_ > RC_SL_ > RC_SN_ > CF, while the RC_SB_ and RC_SL_ were 26.3 and 12.2 mm greater than RC_SN_, respectively. Compared with CF, the average soil temperature was significantly (*P* < 0.05) higher by 3.1, 1.7, and 1.5°C under the RC planting treatments (RC_SB_, RC_SL_, and RC_SN_) during each year, respectively. The average ET rate of RC treatments were all lower than CF in each experimental year, and the average decreased by 8.0% (*P* < 0.05). The average yields with RC_SB_, RC_SL_, and RC_SN_ increased by 2,665, 1,444, and 1,235 kg ha^−1^, respectively, and the water use efficiency (WUE) increased by 51.6, 25.6, and 21.1%, compared with CF. RC_SB_ obtained the highest economic benefit, the average net income was higher than CF by 4,020 Yuan ha^−1^. In conclusion, we found that RC planting with biodegradable film mulching in the furrows is the best cultivation pattern in the semi-humid areas of China in terms of both environmental and economic benefits.

## Introduction

In China, the semi-humid region with annual precipitation of 400–600 mm accounts for 13.5% of the total dry land farming area. Agricultural production in this region is important for food safety given the current shortage of water resources and continuous increases in the population. However, over 70% of the precipitation is distributed in the summer season and < 40% of the total precipitation can be used by crops (Wang et al., 2004; Sun et al., 2016). The seasonal water deficit and low water use efficiency (WUE) are now widespread issues that affect agricultural production (Yao and Yin, 1999; Ren et al., 2017).

Mulching is regarded as one of the best ways for improving water retention in the soil (Wang et al., 2009) and reducing soil evaporation (Li et al., 2008). In recent years, several mulching techniques have been developed and adopted in northwest China, including (1) alternating ridges and furrows with only the ridges mulched with plastic film (RC system) (Li et al., 2016b; Ren et al., 2016), (2) alternating mulched rows and bare rows without ridges (Zhang et al., 2017), and (3) flat plots mulched with plastic film (Li et al., 2010). It is known that different mulching techniques provides different effects on crop growth environment. Rainfall harvesting (RC) system are employed widely to harvest rainwater in-situ in the semi-humid areas of China (Zhang et al., 2007). Compared with conventional flat planting, the amount of daily solar radiation received can be raised by 10–90% using the RC system, which obviously increases the soil temperature (Tian et al., 2003; Gan et al., 2013). Thus, the crop yield is improved significantly due to the better hydrothermal conditions (Zhou et al., 2009; Ren et al., 2016), but the effects on the grain yield were not significant and it even decreased in a rainy year (annual rainfall >440 mm) due to low temperature causing high soil water storage levels in the topsoil (0–40 cm). In recent years, another developed and widely adopted technique is double ridges and furrows mulched with plastic film (Zhou et al., 2009, 2012). Studies have shown that, compared with RC planting, use of this technique can improve topsoil temperature (0–60 cm), especially at the seedling stage, increase topsoil moisture, improve WUE, and enhance crop (maize, potato) yield (Zhou et al., 2009; Zhao et al., 2014). However, its widespread use has generated large amounts of mulch residue, which is called “white pollution” and leads to unsustainable farmland use.

Hence, it is necessary to investigate the effects of furrow mulching with environment-friendly materials under RC system on soil WUE and crop yield. This pattern could decrease the plastic film amount by 50% compared to double ridges and furrows planting, which can alleviate the “white pollution” significantly. In addition, it had a significant effect on water harvesting, improved soil temperature, increased crop yields and produced a greater economic benefit. Although there has been quite some research on plastic film mulched with both ridges and furrows (Ma et al., 2008; Liu et al., 2009; Zhou et al., 2012; Chen et al., 2013), these studies were mainly concentrated in arid regions that received 200–300 mm of annual rainfall. But this planting pattern may not be entirely suitable for crop production in the semi-humid area. Moreover, plastic film was a major mulching material in past studies on RC planting, and there are fewer results on application of environmentally-friendly materials. In particular, we wanted to compensate for low temperature during the corn seedling stage and for the drought that occurs during the growing season in this region. Therefore, we conducted a 4-year study at the field test station of Northwest A&F University, Heyang County, Shaanxi province, China (a typical semi-humid area), where the planting furrows in the RC planting system were mulched with biodegradable film, liquid film, or left uncovered. The objectives of our study were to: (1) investigate the effects of combining the RC system with mulching different materials in furrows on the soil water content, temperature, and corn yield, to provide a scientific basis for improved rainwater harvesting planting system; and (2) compare and analyze the economic benefit, and single out an optimum RC system pattern for semi-humid areas that are prone to drought and low temperatures.

## Materials and methods

### Site description

A field study was conducted from April 2007 to October 2010 at the Ganjing Testing Site of Northwest A&F University, Heyang County, Shaanxi province, China (35°15′N, 110°18′E; 850 m a.s.l.). The site is located in a typical semi-humid area with average annual precipitation of 550 mm, which mainly occurs in July, August, and September. The average annual evaporation was 1,832 mm, the average annual temperature was 11.5°C, the frost–free period was 160–200 days, and the effective accumulated temperature ≥ 10°C was 2,800–4,000°C.

**Figure 1:**
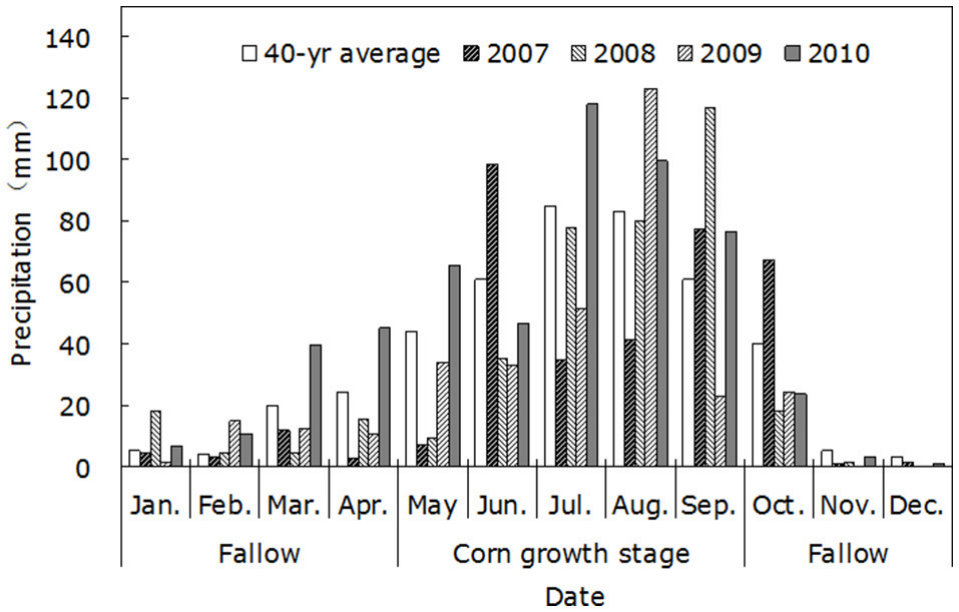
Annual precipitation and its distribution in the test fields during 2007–2010.

The soil at the experimental field was a silt loam. The soil water content throughout the 0–200 cm soil layer before the experiment is shown in Table 1. And the key soil nutrient content of the soil layers (0–30 cm depth) in the experiment field is shown in Table 2. From 2007 to 2010, the annual evaporation was 1,854, 1,954, 2,015, and 1,789 mm, the rainfall rates during the crop growth period (from April 15 to August 30) were 372, 330, 337, and 390 mm, respectively. The rainfall distribution during the experimental periods is shown in Figure 1.

**Table 1:** Initial water content throughout the 0–200 cm soil layer before the experiment.

**Soil depth (cm)**	**0–20**	**20–40**	**40–60**	**60–80**	**80–100**	**100–120**	**120–140**	**140–160**	**160–180**	**180–200**
Water content (%)	10.9	14.2	16.4	16.0	16.2	16.6	17.3	17.9	17.8	18.4

**Table 2:** Basic soil nutrient content in the experiment field.

**Soil depth (cm)**	**Organic matter(g kg^−1^)**	**Total nitrogen (g kg^−1^)**	**Total phosphorus (g kg^−1^)**	**Total potassium (g kg^−1^)**	**Available nitrogen (mg kg^−1^)**	**Available phosphorus (mg kg^−1^)**	**Available potassium (mg kg^−1^)**	**pH**
0–10	12.03	0.94	0.68	7.86	94.21	26.45	161.52	8.1
10–20	10.75	0.81	0.62	7.27	74.83	23.85	137.65	8.1
20–30	9.85	0.71	0.49	6.13	54.18	19.28	108.24	8.1
0–30 Average	10.88	0.82	0.60	7.09	74.41	23.19	135.80	8.1

### Experimental design and field management

**Figure 2:**
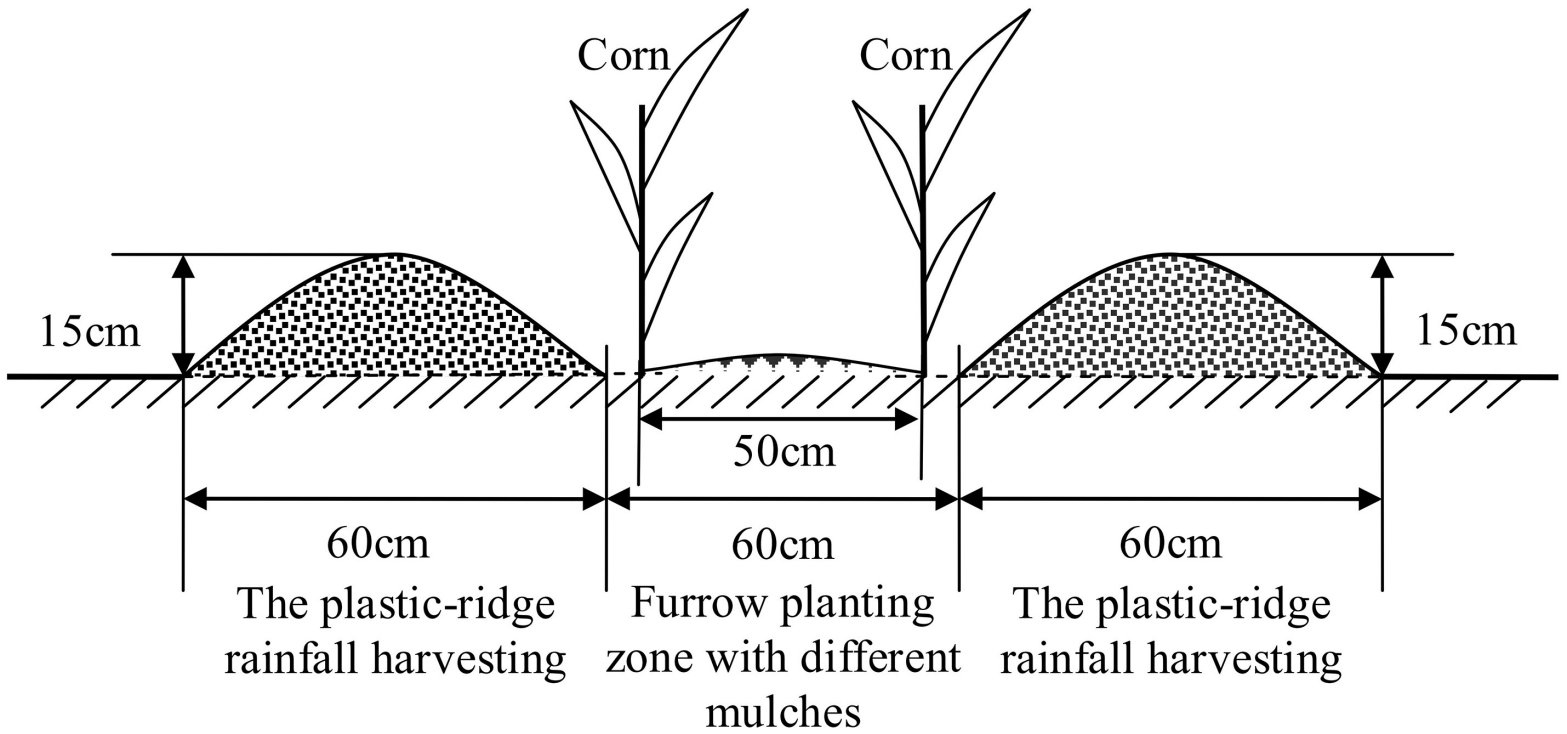
Cross-sectional view of combined RC planting and mulching.

Using the regular local fertilization practices, the field study was performed with RC (rain-harvesting ridges and planting furrows) planting, where the ridges were covered with plastic film (PE film, 0.7 m wide and 0.008 mm thick) and the furrows were mulched with 8% biodegradable film (RC_SB_), which was 0.7 m wide and 0.01 mm thick, liquid film (RC_SL_), or no mulching in the furrows (RC_SN_), whereas conventional flat planting (CF) was employed as the control. The experiment employed a completely randomized design with three replicates (Table 3) and each plot area measured 40 m^2^ (4 × 10 m). The ridge:furrow width ratio of 1:1, and the widths of the ridges and furrows were both 60 cm, the ridge height was 15 cm, and the seeds were sown on the internal sides of the furrows near the ridges (Figure 2). The corn seed cultivar “Yuyu 22” was sown at a rate of 55,600 plants ha^−1^ (60 × 30 cm) at a depth of 5 cm. The plant spacing in the CF and RC treatments was all 30 cm with a row spacing of 60 cm, the row spacing was same with the furrows width that easy to realize mechanization in future. The side rows of each catchment served as protective rows. Plastic films that functioned as water separation belts were buried at a depth of 2 m along the edges of each catchment, thereby preventing water percolation within the soil among catchments.

**Table 3:** Field test treatments.

**Treatments**	**Ridges mulched with materials**	**Furrows mulched with materials**	**Abbreviations for corresponding treatments**
Treatment 1	0.08 mm films	8% biodegradable films	RC_SB_
Treatment 2	0.08 mm films	Liquid films	RC_SL_
Treatment 3	0.08 mm films	No mulching	RC_SN_
Control Group	No ridge and no mulching	No mulching	CF

For the 4 year consecutive field experiment (same treatment in same position in each experimental year), at 30 days before sowing, the ridges were banked up with soil on the spot and covered with plastic film (PE film, 1.0 m wide and 0.008 mm thick), the spring corn was all sown on April 15 during 2007 to 2010, and harvested around August 30 (the date of maturity varied by 3–5 days among different treatments). After harvesting, the configurations and mulches were retained in the same location for all of the plots. The corn stalks were removed, the mulching film was cleared up, and then the furrows were leveled 30 days before subsequent sowing, and the other sowing activities were the same as those in 2007. In order to reduce the experiment influence factors, in each experimental year, a base manure containing 300 kg N ha^−1^, 150 kg P_2_O_5_ ha^−1^, and 150 K_2_O kg ha^−1^ was performed 30 days before sowing. Manure was spread evenly in the CF, whereas it was only applied in the planting furrows under ridge and furrow planting, although the total amounts utilized were the same as those applied to the flat areas.

No irrigation was applied during the entire corn growth period. Weeding and pest control were conducted as necessary. The herbicide (Acetochlor, Jiangsu Wono Chemical Co., Ltd., Jiangsu, China) was sprayed for weed control during the growth period before film mulching. And pesticide (Acephate, Cangzhou Zhongtian Chemical Co., Ltd., Hebei, China) was used for pest control (mainly is corn borer) during the growth period as necessary. The plastic film used in this study was made by Shanxi Yuncheng Plastic Factory and the biodegradable film was supplied by Shaanxi Huayu High-tech and Biological Co. Ltd (the biodegradable film comprised polyethylene and starch, and the degradation process started after 60 days). The liquid film was produced by Zhejiang Aiketai Investment Co. Ltd and it was applied by spraying the mulch evenly over the furrows with a sprayer (biochemical fulvic acid with an expected life span of 40 days applied at a dilution ratio of 1:9, as recommended by the producer).

### Sampling and measurement

The soil water contents were determined gravimetrically to a depth of 200 cm at 20 cm intervals manually in each plot at 0, 30, 60, 90, and 120 days after sowing corn by using a 54 mm diameter steel core-sampling tube, where each sample was replicated three times. The final water determination in the overall corn growing season was performed at the harvest. In ridge-furrow water conservation planting belts, samples were collected from the planting furrows at half of the furrow width, from the boundaries of ridges and furrows, and at half of the furrow width under the ridges. In CF, samples were taken halfway between the planting rows. Soil samples were obtained by auger boring (with a diameter of 0.08 m). Three replicates were taken for each sampling position. The soil samples were weighed wet, dried in a fan-assisted oven at 105°C for 48 h, and weighed again to determine the soil water content.

The soil water storage was calculated using Equation (1):

(1) $$\begin{array}{r} W=h\times p\times b\%\times 10 \end{array}$$

where *W* is the soil water storage (mm), *h* is the soil layer depth (cm), *p* is the soil bulk density (g cm^−3^) in a specific soil layer, and *b%* is the percentage of soil moisture by weight.

The water use efficiency (WUE) was calculated according to the field water balance principle (Ren et al., 2017), as follow:

In ridge and furrow planting areas:

(2) $$\begin{array}{rcl} W_{c} & = & P+E_{r}\frac{n_{1}}{n_{2}}P+\left(W_{1}-W_{2}^{\;}\right) \end{array}$$

(3) $$\begin{array}{rcl} WUE & = & \frac{Y}{W_{c}\times \frac{n_{2}}{n_{1}+n_{2}}} \end{array}$$

In flat planting areas:

(4) $$\begin{array}{rcl} W_{c} & = & \left(W_{1}-W_{2}^{\;}\right)+P \end{array}$$

(5) $$\begin{array}{rcl} WUE\; & = & \;\frac{Y}{W_{c}} \end{array}$$

Where *W*_*C*_ (mm) is crop water consumption; *WUE* (kg ha^−1^ mm^−1^) is the WUE in fields; *W*_1_ and *W*_2_ (mm) are the water storage levels determined in 0–200 cm soil layer depth in two consecutive sampling dates (the water storage under ridge and furrow planting was calculated as the average water storage values in the ridges and furrows); *E*_*r*_ (%) is the runoff efficiency at the ridge surface relative to the rainfall, i.e., the rain-harvesting efficiency or runoff efficiency, where the average runoff efficiency of the film-mulched ridges was 0.87 (Li and Gao, 2004); *P*(mm) is the total precipitation during the overall corn growth season, *n*_1_and *n*_2_(cm) are the ridge width and furrow width, respectively, and *Y* (kg ha^−1^) is the grain yield obtained from the total area of the ridges and furrows.

Geothermometers were placed in the rain-harvesting furrows and in the middle of the corn sowing rows for CF at a soil layer depth of 5 cm to monitor the soil temperature. After sowing, temperature data were obtained each day for 30 consecutive days. Each daily temperature observation was made from 8:00 a.m. to 8:00 p.m. and the data were recorded once every 2 h. The air temperature was recorded in the surrounding fields at the same time as the soil temperature was recorded.

At harvest, two rows of representative corn were harvested manually from the middle of each treatment with three replicates, then twenty evenly growing corn plants were selected to determine the yield components (including the spike number per unit area, grain number per spike, and hundred-seed weight) and the economic yield of corn was calculated according to these components. The grain yield was determined at a water content of 12% and the finally grain yield was calculated based on the total land area, including the ridges and furrows.

### Statistical analysis

The experimental data were calculated by Microsoft Excel 2003 software. The data was tested by analysis of variance using SAS 2001 (SAS Institute Inc. USA), where the data obtained from each sampling event was analyzed separately. Mean values from treatments were compared based on the least significant difference test (LSD 0.05) if the *F*-tests were significant at a probability level of 0.05. The variations including year, planting systems as well as their interactions were not significant, therefore, the data were not shown in Tables. All figures were prepared using SigmaPlot 10.0.

## Results

### Soil water storage

**Figure 3:**
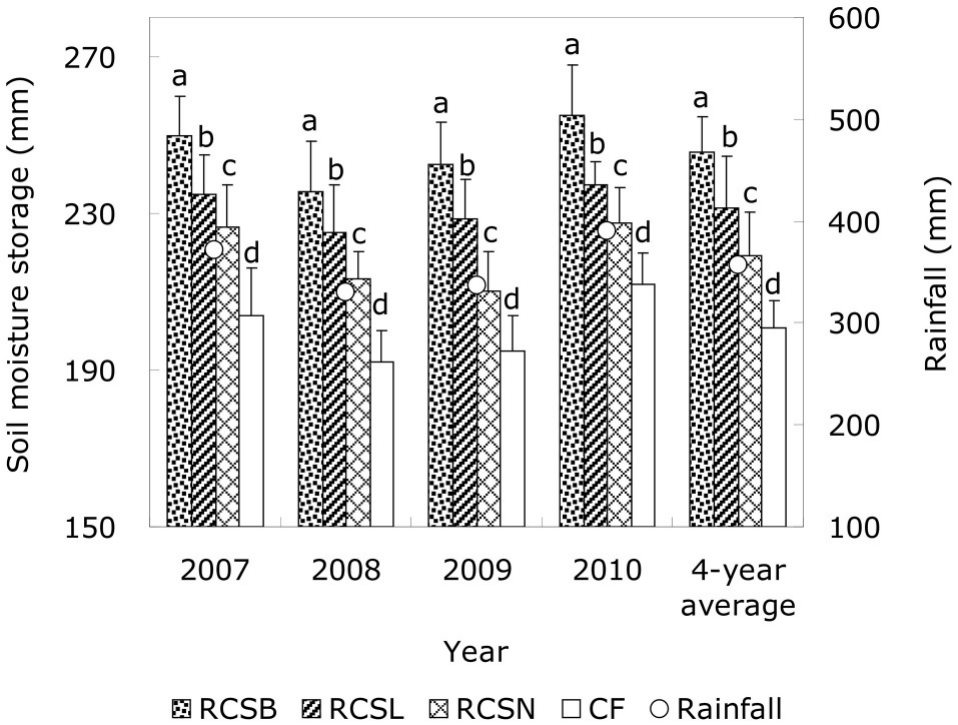
Average field water storage levels in the 0–100 cm soil layer during the growth period of corn from 2007 to 2010. RC_SB_ means rain-harvesting ridges and furrows planting covered with 8% biodegradable films in furrows; RC_SL_ means rain-harvesting ridges and furrows planting covered with liquid films in furrows; RC_SN_ means rain-harvesting ridges and furrows planting with no films mulching in furrows; CF means the conventional flat planting without film mulching. Lowercase letters in the same year indicate significant differences among treatments (LSD test, *P* < 0.05). Bars represent standard deviations.

In four consecutive years, the soil moisture storage was determined regularly in the 0–200 cm soil layer under different treatments during the whole growth period of spring corn. The results showed that the rainfall-harvesting effects of the ridges and furrows significantly improved the soil moisture storage (*P* < 0.05) in the 0–100 cm soil layer under all of the RC treatments compared with conventional flat (CF) farming method, whereas the spatial and temporal changes in moisture storage at depths below 100 cm were relatively small, and there were no significant (*P* < 0.05) differences between the RC treatments and CF; thus, we not show the data of the 100–200 cm depth (Figure 3). The water storage level in the 0–100 cm soil layer differed significantly among test years (*P* < 0.05) with treatments ranking as follows: RC_SB_ > RC_SL_ > RC_SN_ > CF. During 2007–2010, we found that RC_SB_, RC_SL_, and RC_SN_ had the best water-harvesting and moisture-retaining effects; compared with CF, the average soil water storage in the 0–100 cm soil layer with RC_SB_, RC_SL_, and RC_SN_ treatments were significantly (*P* < 0.05) increased by 44.9, 30.7, and 18.6 mm, respectively. The RC planting furrows were mulched with different materials which also have significant (*P* < 0.05) effects on soil water storage. During the crop growth periods in 2007–2010, the annual mean soil moisture storage levels with RC_SB_ and RC_SL_ increased by 26.3 and 12.2 mm, respectively, compared with the RC_SN_ treatment (Figure 3).

### Soil temperature

**Figure 4:**
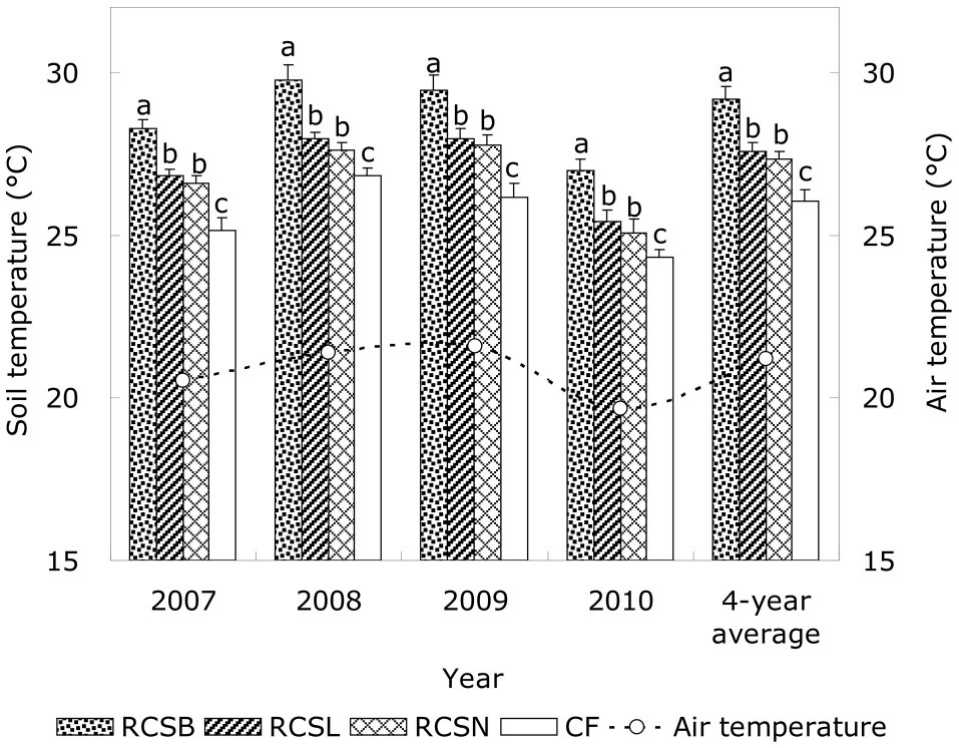
Average soil temperature in the 5 cm soil layer during the early growth period (30 days after sowing) of corn from 2007 to 2010. RC_SB_ means rain-harvesting ridges and furrows planting covered with 8% biodegradable films in furrows; RC_SL_ means rain-harvesting ridges and furrows planting covered with liquid films in furrows; RC_SN_ means rain-harvesting ridges and furrows planting with no films mulching in furrows; CF means the conventional flat planting without film mulching. Lowercase letters indicate significant differences among treatments (LSD test, *P* < 0.05). Bars represent standard deviations.

We found that the soil temperature in the furrows was affected significantly by mulching to depths of 5 cm (*P* < 0.05; Figure 4). The differences among treatments had the same trend in each experimental years as follows: RC_SB_ > RC_SL_ > RC_SN_ > CF. During 2007–2010, the soil temperature was significantly higher under the RC planting treatments than under the CF treatment during each year (*P* < 0.05); under RC_SB_, RC_SL_, and RC_SN_, the average daily soil temperatures over the 4 years were 3.01, 1.43, and 1.15°C higher than that under CF, respectively (Figure 4). Moreover, the RC planting furrows mulched with different materials have different effects on soil temperature; which RC_SB_ had the best warming effects whereas RC_SL_ and RC_SN_ did not perform as well. During the crop growth periods in 2007–2010, the annual mean soil temperature levels with RC_SB_ significantly (*P* < 0.05) increased by 1.58 and 1.85°C, respectively, compared with the RC_SL_ and RC_SN_ treatment (Figure 4). There was no significant difference during each experimental year between RC_SL_ and RC_SN_.

### Grain yield

The results of field experiments in four consecutive years showed that RC planting significantly (*P* < 0.05) enhanced the spring corn grain yield. During 2007–2010, the trend of spring corn yields for each year was similar, and all of the treatments increased the yield (Table 4). Compared with CF, RC_SB_ had the best effects on the yield, followed by RC_SL_ and RC_SN_. Compared with the other mulching treatments, RC_SN_ had a relatively small effect on increasing yield, but compared with CF, the yield still improved by a relatively large amount. The 4-year average grain yields under RC_SB_, RC_SL_, and RC_SN_ increased by 32.3, 17.6, and 14.8% increases compared with CF, respectively. Compared with no mulching in the furrows (RC_SN_), the average annual yields under RC_SB_ and RC_SL_ increased by 17.5 and 2.8%, respectively, and no significant difference during the each experimental year (except 2010, significant higher by 8.6%) between RC_SL_ and RC_SN_.

**Table 4:** Grain yield and field water use efficiency (WUE) during the spring corn growing season in 2007–2010.

**Year**	**Treatment**	**Grain yield (kg ha^−1^)**	**ET (mm)**	**WUE (kg mm^−1^ ha^−1^)**	**Yield increase (%)**
2007	RC_SB_	11276a	472.1c	23.88a	28.9
	RC_SL_	10296b	505.2b	20.38b	17.7
	RC_SN_	10183b	503.5b	20.22b	16.4
	CF	8748c	543.8a	16.09c	—
2008	RC_SB_	11847a	504.4c	23.49a	33.9
	RC_SL_	10560b	544.8b	19.38b	19.4
	RC_SN_	10601b	553.5b	19.15b	19.8
	CF	8844c	581.7a	15.20c	—
2009	RC_SB_	10756a	521.6c	20.62a	32.2
	RC_SL_	9057b	553.3b	16.37b	11.3
	RC_SN_	8921b	563.9b	15.82b	9.6
	CF	8137c	595.7a	13.66c	—
2010	RC_SB_	9763a	473.1c	20.51a	33.7
	RC_SL_	8845b	515.1b	17.17b	21.9
	RC_SN_	8217c	529.4a	15.52c	13.3
	CF	7255d	539.0a	13.46d	—
Average value over 4 years	RC_SB_	10911a	479.9c	22.13a	32.3
	RC_SL_	9690b	529.6b	18.33b	17.6
	RC_SN_	9481b	537.6b	17.68b	14.8
	CF	8246c	565.0a	14.60c	—

*ET, evapotranspiration; WUE, water use efficiency; RC_SB_, means rain-harvesting ridges and furrows planting covered with 8% biodegradable films in furrows; RC_SL_, means rain-harvesting ridges and furrows planting covered with liquid films in furrows; RC_SN_, means rain-harvesting ridges and furrows planting with no films mulching in furrows; CF, means the conventional flat planting without film mulching. Lowercase letters indicate significant differences among treatments (LSD test, P < 0.05). The grain yield refers to the grain yield obtained from the total area of the ridges and furrows*.

### ET and WUE

Similar to the yields, the results of field experiments in four consecutive years showed that RC planting significantly (*P* < 0.05) enhanced the WUE (Table 4). The WUE with four treatments during each experimental years were ranked in the following order: RC_SB_ > RC_SL_ > RC_SN_ > CF, and the average WUE of RC treatments were all significantly (*P* < 0.05) higher than CF, i.e., increased by 33.6% in 2007, 36.0% in 2008, 28.9% in 2009, and 31.7% in 2010. Comparing between the different RC treatments it can be found that: the average WUE with RC_SB_ were significantly (*P* < 0.05) higher than RC_SL_ and RC_SN_ by 20.7 and 25.2% during 2007–2010, respectively. There were no significant difference between RC_SL_ and RC_SN_ during 2007–2009, but RC_SL_ was significant (*P* < 0.05) higher than RC_SN_ by 12.3% in 2010.

During 2007–2010, the trend of *ET*-value of spring corn for each year was similar, and all of the treatments decreased the ET rate (Table 4). The average ET rate of RC treatments were all lower than CF in each experimental years (Table 4), i.e., decreased by 9.2% (*P* < 0.05) in 2007, 8.2% (*P* < 0.05) in 2008, 8.3% (*P* < 0.05) in 2009, and 6.1% in 2010. Comparing between the different RC treatments it can be found that: the average ET rate with RC_SB_ were significantly (*P* < 0.05) lower than RC_SL_ and RC_SN_ by 6.4 and 7.8% during 2007–2010, respectively, and no significant difference during the each experimental year (except 2010, RC_SL_ significant lower than RC_SN_ by 2.7%) between RC_SL_ and RC_SN_.

### Economic benefits

There were obvious differences in the input costs of the various mulching materials treatments, because of the use of different mulching materials (Table 5). The 4-year average input cost was ranked as follows: RC_SL_ > RC_SB_ > RC_SN_ > CF, and the input value under RC_SB_, RC_SL_, and RC_SN_ were 1,710, 1,785, and 885 Yuan ha^−1^ more than CF. The most important output value from the plots was the grain yield, whose market price (local price) was stable between and within experimental seasons. In our study, similar to the grain yield, the output value with the different treatments followed the order: RC_SB_ > RC_SL_ > RC_SN_ > CF, compared with CF, from 2007 to 2010, the average yields under RC_SB_, RC_SL_, and RC_SN_ improved by 2,665, 1,444, and 1,235 kg ha^−1^, respectively, and these greater yields increased the net income of farmers by 3,086, 813, and 1,337 Yuan ha^−1^ (Table 5). The net income of RC_SB_ and RC_SN_ treatments were all higher than that of CF during 2007–2010, average increased by 3,086 and 1,337 CNY ha^−1^, respectively, and due to the high input value, the RC_SL_ treatment was lower than CF by 129 CNY ha^−1^ in 2009.

**Table 5:** Average economic benefits generated by RC planting of spring corn between 2007 and 2010.

**Year**	**Treatments**	**LC^A^**	**MC**	**MCC**	**SFC**	**IV**	**OV**	**YI**	**NI**	**NID**
2007	RC_SB_	1,800	1,260	1,500	3,200	7,760	20,297	2,528	12,537	2,840
	RC_SL_	1,800	1,335	1,500	3,200	7,835	18,533	1,548	10,698	1,001
	RC_SN_	1,650	585	1,500	3,200	6,935	18,329	1,435	11,394	1,698
	CF	1,350	0	1,500	3,200	6,050	15,746	-	9,696	-
2008	RC_SB_	1,800	1,260	1,500	3,200	7,760	21,325	3,003	13,565	3,695
	RC_SL_	1,800	1,335	1,500	3,200	7,835	19,008	1,716	11,173	1,304
	RC_SN_	1,650	585	1,500	3,200	6,935	19,082	1,757	12,147	2,278
	CF	1,350	0	1,500	3,200	6,050	15,919	-	9,869	-
2009	RC_SB_	1,800	1,260	1,500	3,200	7,760	19,361	2,619	11,601	3,004
	RC_SL_	1,800	1,335	1,500	3,200	7,835	16,303	920	8,468	−129
	RC_SN_	1,650	585	1,500	3,200	6,935	16,058	784	9,123	526
	CF	1,350	0	1,500	3,200	6,050	14,647	-	8,597	-
2010	RC_SB_	1,800	1,260	1,500	3,200	7,760	17,573	2,508	9,813	2,804
	RC_SL_	1,800	1,335	1,500	3,200	7,835	15,921	1,590	8,086	1,077
	RC_SN_	1,650	585	1,500	3,200	6,935	14,791	962	7,856	847
	CF	1,350	0	1,500	3,200	6,050	13,059	-	7,009	-
Average value over 4 years	RC_SB_	1,800	1,260	1,500	3,200	7,760	19,639	2,665	11,879	3,086
	RC_SL_	1,800	1,335	1,500	3,200	7,835	17,441	1,444	9,606	813
	RC_SN_	1,650	585	1,500	3,200	6,935	17,065	1,235	10,130	1,337
	CF	1,350	0	1,500	3,200	6,050	14,843	-	8,793	0

*RC_SB_, means rain-harvesting ridges and furrows planting covered with 8% biodegradable films in furrows; RC_SL_, means rain-harvesting ridges and furrows planting covered with liquid films in furrows; RC_SN_ means rain-harvesting ridges and furrows planting with no films mulching in furrows; CF, means the conventional flat planting without film mulching; LC, Labor costs (Chinese yuan (CNY) ha^-1^); MC, film mulching costs (CNY ha^-1^); MCC, machine-cultivated costs (CNY ha^-1^); SFC, seed and fertilizer costs (CNY ha^-1^); IV, input value (CNY ha^-1^) = LC +MC+MCC+SFC; OV, output value (CNY ha^-1^); YI, average yield increase in 4 Years (kg ha^-1^); NI, net income (CNY ha^-1^) = OV – total IV; NID, net income difference (CNY ha^-1^) from CF. ^A^Labor cost was 60 CNY per person per day; plastic film cost was 13 CNY kg^-1^; biodegradable film 15 CNY kg^-1^; liquid film 10 CNY kg^-1^; corn seed price was paid local Chinese government by 1.8 CNY kg^-1^*.

## Discussion

RC planting is regarded as one of the best ways of improving water retention in the soil and reducing soil evaporation (Ren et al., 2010; Li et al., 2013). Many studies have indicated that RC planting leads to significant water conservation and reduced soil water evaporation by mulching on ridges, in turn increasing precipitation use efficiency in rain-fed farming systems (Ramakrishna et al., 2006; Gan et al., 2013). Favorable soil water conditions are vital for obtaining high yields in dry land farming (Li et al., 2010; Zhou et al., 2011). Our results showed that the water storage level was significantly higher in the 0–100 cm soil layer under the RC treatments (*P* < 0.05) compared with conventional CF planting, which was mostly because of the following two possible reasons: one is the water conservation effects of the RC system in which the ridge and furrow system could accumulate rainfall to enhance water infiltration and retention, and another is the plastic film mulching on ridges could significant prevent the soil water exchange between the soil and air to decrease the evaporation of soil water. The current study also showed that there were no significant differences in the water storage levels among different treatments in the soil layer below 100 cm (data was not shown). These results agree with those of our previous study, where we used movable rain shelters to obtain different rainfall during the crop growing season (Ren et al., 2010). The water storage in the 0–100 cm soil layer varied according to the precipitation in different years. Gan et al. (2013) and Li et al. (2013) observed that mulching materials in the RC planting furrows probably reduced evaporation from the soil surface, thereby retaining more water in the soil. In our study, with the RC treatments, the average water storage level in the 0–100 cm soil layer differed significantly among test years (*P* < 0.05) with RC_SB_, RC_SL_, and RC_SN_ as follows: RC_SB_ > RC_SL_ > RC_SN_, during the corn growth period (Figure 3). It indicating that mulching the furrows with biodegradable film and liquid film further enhanced the rain-harvesting and moisture-retaining effects of the RC system, because furrows mulched with different materials inhibited soil evaporation in the RC_SB_ and RC_SL_ treatment but some soil moisture might have evaporated directly from the flat surface of the film thereby preventing the infiltration of light rain (Li et al., 2016a).

Temperature is one of the major factors limiting agricultural productivity (Zhou et al., 2012), and soil temperature is the basic for crops to adapt the various temperatures, which is also an important factor to maintain root activity, change root morphology to affecting crop yield (Stone et al., 1999). Several investigators have reported that suitable temperatures during the early stage of crop growth can greatly accelerate grain germination and dry matter accumulation (Wang et al., 2005; Liu et al., 2010; Imran et al., 2013). The growth of spring corn may be influenced significantly when the soil temperature is below 15°C (Verheul et al., 1996), while growth can be inhibited when the soil temperature is below 8°C (Raes et al., 2009). In the present study, the soil temperature was significantly higher under the RC planting treatments than under the CF treatment during each experimental year (*P* < 0.05), which provided favorable thermal conditions for seed germination and plant growth during the early stages. Mulching influenced the exchange of heat between the soil surface and the atmosphere, thereby greatly affecting the soil temperature. A thin layer of black film formed on the soil surface after the furrows were sprayed with the liquid film, which was more favorable to the absorption of solar energy and this led to a higher soil heat flux (Yang et al., 2005). We observed that the soil temperature under RC_SL_ was slightly higher than the RC_SN_, and no significant difference in the diurnal temperature change between RC_SL_ and RC_SN_. This was because the black liquid membrane was sprayed on soil surface was made the diurnal temperature increase relatively rapidly as the environmental temperature rose but it also decreased relatively rapidly as the temperature declined. Our study also showed that the average daily soil temperatures was higher under RC_SB_ was significant higher than RC_SL_ and RC_SN_, and the improvement in the soil temperature was greater with the biodegradable film than that with liquid film (Subrahmaniyan and Zhou, 2008; Chen et al., 2013). This was a result of two mechanisms: the biodegradable polymer film mulch had an air gap between the film and the soil surface, while the sprayed liquid membrane had direct contact with the soil (Schettini et al., 2007; Immirzi et al., 2009). These results indicate that the topsoil temperature was effectively improved by RC planting and the warming effects were enhanced further by mulching the furrows with biodegradable film, which provided highly favorable conditions for seedling growth in the experimental area.

Previous studies showed that the crop yield is positively correlated with the ET rate (Sun et al., 2006). However, we got a different result because of the different environment and planting management in our continuous field experiment. Our study showed that the average ET rate of RC treatments were all lower than CF in each experimental years (Table 4). Because the RC planting could significantly reduced soil water evaporation and improved water retention (Ren et al., 2016; Zhang et al., 2017), then increased soil water storage in the maturing stage, in consequence, the ET rate was lower with RC treatments than CF. Our study also showed that in each experimental year, the ET rate of RC_SB_ were lower than RC_SL_ and RC_SN_. And no significant (*P* > 0.05) difference between RC_SL_ and RC_SN_, which was consistent with Zhang et al. (2010) results. Because the soil water evaporation under RC_SL_ and RC_SN_ significantly higher than that under RC_SB_, which caused by the liquid film was easy to damaged by environmental conditions after spraying in field (Li et al., 2013).

Water use efficiency (WUE) is a comprehensive index that represents the relationship between water consumption and the grain yield (Turner, 1986). Thus, it is commonly used to develop and evaluate optimum water management strategies to ensure the most efficient use of water resources (Liu et al., 2010). Previous studies have shown that the ridge and furrow system planting can have major effects on the field WUE by affecting the soil water content and soil temperature (Wang H. L. et al., 2011; Gan et al., 2013). In our present study, the results of 4-year consecutive field tests showed that the average WUE of RC treatments were all significantly (*P* < 0.05) higher than CF in each experimental years, this was because the surface mulch favorably influences the soil moisture regime by controlling evaporation from the soil surface (Jia et al., 2006; Wang et al., 2009), improving infiltration and soil water retention, support a favorable soil microclimate for crop growth and root proliferations (Li et al., 2012), and thus the water passing through the crop by transpiration increased (Jia et al., 2006). These agree with the results reported Song et al. (2013) who studied the effects of field ridging at different times on the corn yield in the dryland farming area of northeast China at high latitudes. Due to the favorable soil moisture and temperature conditions for spring corm under the rain-harvesting planting system. Our study also showed that, compared with RC_SL_ and RC_SN_, the RC_SB_ treatment significantly (*P* < 0.05) increased the field WUE by 20.7% and 25.2% during 2007–2010, respectively, which indicates that mulching the furrows with biodegradable film can greatly enhance the crop transpiration under rain-harvesting planting. It was possibly because the RC_SB_ treatment could reduce the area available for evaporation from the fields, decrease ineffective evaporations from the soil, and thus the RC_SB_ treatment obtained a higher WUE than other treatments (Shen et al., 2011; Jia et al., 2017). Wang M. et al. (2011) reported that the application of liquid film mulch could increased the maize yield and WUE. However, we found no differences in the WUE with the RC_SL_ and RC_SN_ treatments, which may be related to the composition of liquid film or it may suggest that the film was more vulnerable to the environmental conditions that prevailed during film formation after spraying (Zhang et al., 2010).

Mulching had significant effects on the soil temperature and water content, which affected the crop growth and yield (Turner et al., 2011). Many studies had showed that the film mulching can improve the soil water temperature conditions during the early growth stage, promote the crop vegetative growth, thus in favor of the crop reproductive growth to increase the grain yield (Liu et al., 2010; Gan et al., 2013). Wang et al. (2016) recommend that plastic-film mulch can be preferentially applied for maize production in semiarid environments where both insufficient rainfall and low temperature are simultaneous limiting factors after assessment the effects of RC system in multi-site in semiarid areas. Our study showed that the corn grain yields in the RC treatments were all significantly (*P* < 0.05) higher than CF in each experimental year, which agreed with the results of previous studies conducted in dry land farming in semi-arid areas (Zhou et al., 2012; Song et al., 2013). This was mostly because the RC planting can prolong the period of moisture availability and provide a optimum soil temperature by improve soil moisture storage and topsoil temperature (Wang H. L. et al., 2011; Li et al., 2013; Ren et al., 2016), thereby enhance production of agricultural crops. Comparing between the different RC treatments can be found that: RC_SB_ treatment achieved a higher yield compared with RC_SL_ and RC_SN_ (Table 4), which indicates that mulching the furrows with biodegradable film can greatly enhance the yield under ridge-furrow water conservation planting. Moreno and Moreno (2008) reported that although biodegradable film underwent early decomposition, but it generally remained functional during use and it did not affect the yield increase, which was consistent with our results. Due to the special composition, the liquid film might have been more vulnerable to the environmental conditions that prevailed during film formation after spraying (Qiang et al., 2010; Li et al., 2012). In our study, the differences between RC_SL_ and RC_SN_ were not significant significantly (*P* > 0.05), except in 2010 when the air temperature during the spring was relatively low, and the superior effect of RC_SL_ on increasing the soil temperature was highlighted.

Economic benefit is one of the most effective evaluation indexes for crop management practices, which is the most concerned by farmers. The most important output from the plots was corn grain, the market price of which is stable between and within seasons in northwest China. We found that compared with CF, RC treatments significantly increased the OV because the corn grain yield (14.8–32.3%) significantly increased, which also showed by Li et al. (2013, 2016b). Our study also found that the input value (IV) for RC treatments were all higher than CF and ranked as follow: RC_SL_ > RC_SB_ > RC_SN_ > CF, because the mulching materials and labor costs were different. Comparing between the different RC treatments can be found that the RC_SB_ treatments could significantly increased NI compared with RC_SL_ and RC_SN_, this mainly because furrows mulching with biodegradable film could provide a favorable soil water temperature conditions for corn growth to produced a higher grain yield. Therefore, the utilization of RC planting combined with biodegradable film mulching in the furrows can greatly improve the crop yields and enhance the revenues for farmers in the semi-humid farming areas of China.

## Conclusion

The results of this study demonstrate that in rain-harvesting planting, plastic film mulching on ridges and furrows mulching with 8% biodegradable film (RC_SB_ treatment) can inhibit soil evaporation, improve the soil moisture storage and availability in the furrow, regulate the soil temperature, as well as improving corn yield and field WUE, thereby obtaining greater economic benefits for local farmers. Therefore, farmers will achieve greater grain production if this method is applied as an efficient cultivation pattern in the semi-humid dryland farming areas of the Loess Plateau in China, and possibly in other similar areas.

## Author contributions

The manuscript was reviewed and approved for publication by all authors. XR, ZJ, and PZ conceived and designed the experiments. PZ, XC, TC, TW, XR, and YW performed the experiments. PZ, TW, TC, and ZJ analyzed the data. PZ, XC, and XR wrote the paper. XR, XC, PZ, TW, TC, YW, SA, and ZJ reviewed and revised the paper. PZ, TW, and ZJ corrected the English language for the paper.

### Conflict of interest statement

The authors declare that the research was conducted in the absence of any commercial or financial relationships that could be construed as a potential conflict of interest.
